# Retraction Note: Ultrasound versus temporal artery biopsy in patients with Giant cell arteritis: a prospective cohort study

**DOI:** 10.1186/s12880-020-00454-7

**Published:** 2020-05-21

**Authors:** Quan Zou, Sumei Ma, Xinghu Zhou

**Affiliations:** 1 Department of Ultrasound, the first hospital of Lanzhou University, Lanzhou, 730000 China; 2 Department of Cardiology, the first hospital of Lanzhou University, Lanzhou, 730000 China

**Retraction to: BMC Medical Imaging (2019) 19:47**

**https://doi.org/10.1186/s12880-019-0344-2**

The Editor has retracted this article [1] because Figure 1 appears to have multiple duplications and replications throughout. This article is therefore unreliable. Quan Zou does not agree with this statement. Sumei Ma and Xinghu Zhou have not responded to any correspondence from the Editor about this retraction.
